# Amivantamab: A Novel Advance in the Treatment of Non-small Cell Lung Cancer

**DOI:** 10.7759/cureus.60851

**Published:** 2024-05-22

**Authors:** Esteban Zavaleta-Monestel, Jonathan García-Montero, Sebastián Arguedas-Chacón, Ricardo Quesada-Villaseñor, Monserrat Barrantes-López, Rebeca Arroyo-Solís, Carlos E Zuñiga-Orlich

**Affiliations:** 1 Pharmacy, Hospital Clínica Bíblica, San Jose, CRI; 2 Pharmacy/Research, Hospital Clínica Bíblica, San José, CRI; 3 Pharmacy/Research, Hospital Clínica Bíblica, San Jose, CRI; 4 Medical Research, Hospital Clínica Bíblica, San José, CRI; 5 Pharmacy, Universidad de Iberoamérica, San José, CRI; 6 Oncology, Hospital Clínica Bíblica, San José, CRI

**Keywords:** monoclonal antibody, exon 20, amivantamab, epidermal growth factor receptors, non-small cell lung cancer

## Abstract

Amivantamab is a fully human bispecific monoclonal antibody indicated for treating patients with specifically large cell lung cancer. Its dosage is based on the patient's initial body weight and is administered via intravenous infusion after dilution. Therefore, this drug is given as a strategy due to the great need for a molecule targeting epidermal growth factor receptor (EGFR) and the mesenchymal-epithelial transition factor (MET), as acquired resistance to tyrosine kinase inhibitors (TKIs) was observed in the treatment of large cell lung cancer. This article encompasses a review of the benefits of amivantamab for patients with non-small cell lung cancer (NSCLC). This drug is the first therapy directed against this specific mutation, and unlike others, it could bind to two genetic receptors, whereas antibodies, in general, are directed toward a single receptor.

## Introduction and background

Every day, new cases of cancer arise worldwide. According to the latest Global Cancer Observatory (GLOBOCAN) estimates, in 2018, 2,094,000 new cases of lung cancer were diagnosed, making it the most common cancer globally. With an estimated 1,369,000 cases, lung cancer ranks as the second most common cancer in men after prostate cancer, and it is the second most common cancer in women after breast cancer, with 725,000 cases [1].

Lung cancer and all its histological subtypes are more prevalent in high-income countries, primarily due to tobacco consumption. The specific incidence of small cell lung cancer is not well-defined; however, it is considered more prevalent in men than in women, although over the past 50 years, the proportion of cases in women compared to men has been increasing [2].

Non-small cell lung cancer (NSCLC) is a complex disease that requires in-depth understanding for research and treatment. This type of cancer is characterized by the formation of cancerous (malignant) cells in different lung tissues. There are various types of NSCLC, and the primary risk factor is smoking. In fact, being an active smoker is the main risk factor for developing this disease. When NSCLC is suspected, various tests and lung biopsies are performed to determine the stage and type of cancer. This process is crucial for accurate diagnosis and appropriate treatment [3,4].

The symptoms of non-small cell lung cancer (NSCLC) are often nonspecific in the early stages, making early detection challenging. Some patients may experience symptoms such as weight loss, hyporexia (decreased appetite), and asthenia (weakness). Patients with NSCLC metastasis may experience specific symptoms in the affected organs. The most common metastatic sites are the brain, liver, and bones. Brain metastases can cause headaches, seizures, and neurological changes. Liver metastases can cause abdominal pain, nausea, and vomiting. Bone metastases can cause bone pain, fractures, and bone fragility, among others [3].

Non-small cell lung cancer (NSCLC), like many other types of cancer, presents alterations in the DNA sequence (mutations) and abnormalities in gene expression. These abnormalities, which typically originate in a single cell, can activate oncogenes, and deactivate tumor suppressor genes and DNA repair genes. The most common mutation in NSCLC is in the epidermal growth factor receptor (EGFR) [5]. This mutation allows tumor cells to grow and survive independently of external signals, turning them into cancer cells. There are other molecular mechanisms involved in the development of NSCLC, such as those affecting receptors and transducers of biochemical signals and tumor suppressor genes. Among the most important genes in this regard are P53 and DNA repair genes. Currently, various molecular markers are being studied in NSCLC, such as epidermal growth factor receptor (EGFR), Kirsten rat sarcoma viral oncogene (KRAS), excision repair cross-complementation 1 (ERCC1), ribonucleotide reductase large subunit M1 (RRM1), vascular endothelial growth factor (VEGF), anaplastic lymphoma kinase (ALK), and mesenchymal-epithelial transition factor (MET). These markers can help diagnose the disease, determine its prognosis, and select the most appropriate treatment for each patient [3].

This review focuses on studying the benefits of the drug amivantamab, also known by its brand name Rybrevant, which is indicated for the treatment of non-small cell lung cancer. Rybrevant is a fully human bispecific monoclonal antibody directed at the EGFR and MET receptors and indicated for the treatment of adult patients with specifically large cell lung cancer. It is administered at a dosage based on the patient's initial body weight, then delivered to the patient as an intravenous infusion after dilution of the medication. The purpose of this review is to inform and assist patients with this condition, as well as their families and caregivers, by letting them know that there is a medication that has shown lasting response in patients with this disease, for which currently there are no targeted and approved treatments [6].

## Review

Drug history

This bispecific antibody is presented as an alternative due to the need for a molecule targeting the EGFR and MET receptors, as acquired resistance to tyrosine kinase inhibitors (TKIs) was observed. The initial objective was to develop a strategy to target both pathways. To achieve this, a platform was utilized to enable the use and exchange of antigen-binding fragments. Through multiple rounds of testing, an antibody with the desired characteristics was obtained, including high cytotoxic capacity and low fucose content, giving rise to amivantamab [6].

In May 2021, amivantamab received approval from the United States Food and Drug Administration (FDA) for the treatment of patients with locally advanced or metastatic lung cancer with EGFR Ex20Ins mutations. This approval was based on the results of the CHRYSALIS clinical trial, a multicenter, non-randomized, open-label phase I study. The CHRYSALIS trial demonstrated that amivantamab was effective in reducing tumor size and improving survival in patients with lung cancer with EGFR Ex20Ins mutation [7]. Table 1 shows the anti-EGFR antibodies approved for the treatment of lung cancer between 2004 and 2021, where it is demonstrated for different reasons that amivantamab is the best treatment option [8].

**Table 1:** Approved anti-EGFR antibodies for the treatment of lung cancer SCCHN - head and neck squamous cell carcinoma; OS - overall survival; ORR - objective response rate; CR - colorectal cancer; PFS - progression-free survival; DOR - duration of response; EGFR - epidermal growth factor receptor; NSCLC - non-small cell lung cancer; MET - mesenchymal-epithelial transition; MOA - mechanism of action; ADCC - antibody-dependent cellular cytotoxicity; ADCP - antibody-dependent cellular phagocytosis; ADCR - antibody-dependent cytokine release; ADCT - antibody-dependent cellular trogocytosis; CRC - colorectal cancer

Year of initial approval in the US	2004	2006	2015	2021
Generic name	Cetuximab	Panitumumab	Necitumumab	Amivantamab
Brand name	Erbitux	Vectibix	Portazza	Rybrevant
Company	Eli Lilly	Amgen	Eli Lilly	Jansen
Indication	SCCHN: In combination with radiation therapy, platinum-based therapy with fluorouracil, or after progression of platinum-based therapy.	Metastatic colorectal cancer with disease progression during or after chemotherapy regimens with irinotecane, oxaliplatin, and fluoropyrimidine	Gemcitabine and cisplatin for metastatic squamous NSCLC	Lung cancer (NSCLC) with a specific genetic mutation (EGFR ex20ins) that has worsened despite receiving platinum-based chemotherapy.
	Metastatic CRC with EGFR expression and KRAS wild-type: In combination with FOLFIRI in combination with irinotecan in patient’s refractory to irinotecan-based chemotherapy; as a single agent in patients who have failed oxaliplatin- and irinotecan-based chemotherapy or who are intolerant to irinotecan.			
Design (schematic)	Recombinant human/mouse chimeric AcM IgG1	Recombinant human IgG2 monoclonal antibody.	Recombinant human IgG1 monoclonal antibody.	Anti-MET bispecific IgG1 and human anti-EGFR
MOA	Ligand blockade, ADCC	Ligand blockade.	Receptor degradation, ADCC	Ligand blockade, receptor degradation, ADCC, ADCP, ADCR, ADCT.
Common adverse events (≥30%), all grades	Skin adverse reactions (including rash, pruritus, and nail changes), headache, diarrhea, infection.	Acneiform dermatitis (57%), itching (57%), erythema (65%), skin toxicity (90%), hypomagnesemia (38%),	Hypophosphatemia (31%), hypocalcemia (45%), rash (44%), hypomagnesemia (83%),	Fatigue (33%), musculoskeletal pain (47%), nausea (36%), rash (84%), paronychia (50%), dyspnea (37%),
Efficacy	Among 329 patients with CRC + irinotecan; ORR: 23%; DOR: 5.7 months.	Among 231 patients: PFS: 96	Among 545 patients: OS: 11.5 months PFS: 5.7 months	Among 81 patients: ORR: 40% DOR: 11.1 months
	Among 387 patients with previously treated CRC; OS: 6.1 months			
	Among 222 patients with SCCHN + platinum-based therapy + fluorouracil; ORR: 35.6%.			
	Among 608 patients with CRC + FOLFIRI; ORR: 46%.			

Mechanism of actions

Mutations are very common in NSCLC, the third most common type being EGFR Ex20Ins mutations, which have variable insertion points found between codons 767-774, thus collectively representing 4 to 10% of mutations in EGFR. The EGFR Ex20Ins mutations exhibit a unique structural configuration that distinguishes them from typical EGFR mutations. This peculiarity renders them relatively resistant to EGFR tyrosine kinase inhibitors (TKIs), the drugs commonly employed to treat non-small cell lung cancer (NSCLC) [7].

EGFR mutations function as a faulty switch that triggers aberrant cellular signaling, promoting uncontrolled cancer growth. Compared to normal EGFR, mutant receptors exhibit a lower affinity for ATP, the molecule that fuels their activity. This disparity enables tyrosine kinase inhibitors (TKIs), drugs employed to treat cancer, to compete with ATP and block abnormal signaling. However, EGFR Ex20Ins mutations exhibit a unique configuration that renders them more resistant to TKIs. These mutations retain the ability to bind ATP but simultaneously adopt a more rigid conformation at the drug-binding site, hindering TKI interaction [7].

Optimizing treatment for patients with EGFR Ex20Ins-mutant non-small cell lung cancer (NSCLC) presents a significant challenge, often requiring a delicate balance between efficacy and safety. A major hurdle lies in the development of acquired resistance to EGFR tyrosine kinase inhibitors (TKIs). This resistance can stem from mutations within the EGFR protein itself or the acquisition of new mutations in the MET receptor. Both EGFR and MET receptors play crucial roles in tumorigenesis, driving the growth and spread of cancer by promoting oncogenic signaling and fostering a supportive tumor microenvironment. This intricate interplay between the two pathways opens a promising avenue for therapeutic intervention: simultaneously targeting both EGFR and MET could potentially improve clinical outcomes and overcome resistance to existing TKIs [7].

**Figure 1:**
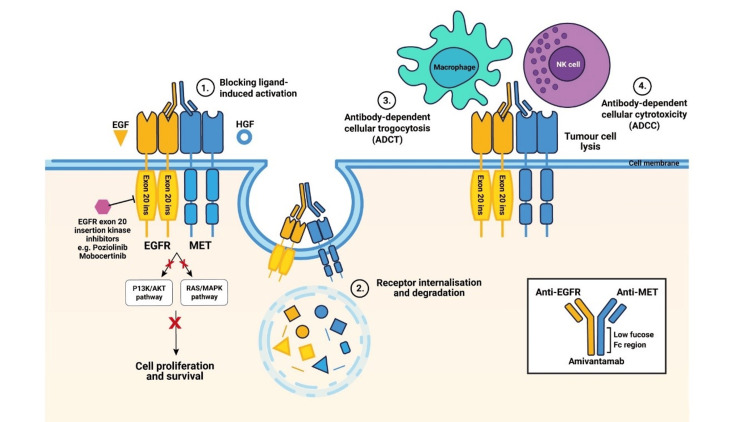
Mechanism of action of amivantamab Adapted from Vyse et al. [10]

Amivantamab is a bispecific antibody that targets both the epidermal growth factor receptor (EGFR) and the mesenchymal-epithelial transition factor (MET). This drug binds to the extracellular domains of EGFR and MET to disrupt their signaling, blocking ligand binding and ultimately inhibiting receptor phosphorylation, particularly in exon 20 insertion mutation models. This leads to the degradation of EGFR and MET. Thus, the presence of EGFR and MET on the surfaces of tumor cells enables immune effector cell-mediated cell destruction, macrophages, and natural killer cells through mechanisms of antibody-dependent cellular cytotoxicity and trogocytosis, respectively [9]. Figure 1 illustrates the mechanism of action of amivantamab [10].

Pharmacodynamics

Amivantamab demonstrates multifaceted activity by inhibiting the signaling of EGFR and MET, which has a high affinity for hepatocyte growth factor (HGF), both cell surface receptors involved in regulating cell growth, survival, and differentiation, through various mechanisms, suggesting significant therapeutic potential in the treatment of cancers associated with these signaling pathways [10].

EGFR and MET or (HGFR) are two additional types of receptor tyrosine kinases. They are involved in non-small cell lung cancer but not in small cell lung cancer (SCLC). Tyrosine kinase inhibitors are commonly used to treat NSCLC, but their effectiveness can diminish due to secondary mutations in the EGFR gene. In such cases, amivantamab, a bispecific monoclonal antibody targeting EGFR and MET, can be a viable treatment option [11].

Amivantamab binds directly to cell surface receptors, preventing them from activating by blocking the attachment of growth-stimulating molecules. The drug binds to receptors, triggering their internalization and destruction within the cell, further reducing their signaling potential. Amivantamab attracts immune cells like macrophages and monocytes, which then engulf small fragments of the tumor cell membrane containing EGFR and MET receptors through a process called antibody-dependent cellular trogocytosis (ADCT). Activated natural killer (NK) cells are able to directly kill tumor cells coated with amivantamab through a process called antibody-dependent cellular cytotoxicity [10].

Pharmacokinetics

The administration route of amivantamab is intravenous. It is administered weekly for a period of four weeks, followed by every two weeks until disease regression is achieved. The pharmacokinetic profile of amivantamab revealed a significant difference in drug exposure (AUC: area under the serum concentration-time curve) between the fifth and first doses. This observation points towards potential drug accumulation with multiple dosing regimens [9].

After two months of biweekly administration, the drug's concentration in the body reached a stable equilibrium state during the ninth infusion, when a dose of 1050 mg was administered. Compared to the initial dose, the average initial area under the curve (AUC1) was higher at this equilibrium point. In fact, it was observed to be 2.4 times higher. Pharmacokinetic analysis revealed that the drug has a distribution volume of 5.13 liters and an elimination half-life of 11.3 days [9].

The clearance of this medication is 360 ml per day. A higher clearance is observed at lower doses, specifically under 350 mg, remaining linear within this therapeutic range. Additionally, both the clearance and the volume of distribution of the medication increase with body weight increment. Regarding exposure to amivantamab, no clinically significant effects related to gender, race, age, presence of mild hepatic impairment, or creatinine clearance were observed [9].

Clinical indications

Amivantamab is a targeted therapy specifically indicated for the monotherapy treatment of locally advanced or metastatic NSCLC in adult patients. This indication applies only to individuals with confirmed activating EGFR exon 20 insertion mutations identified through an approved test, whose disease has progressed despite prior platinum-based chemotherapy. Its approval was granted through an accelerated process based on a favorable relative risk and the observed response duration. However, further clinical trials may be required for its long-term availability [12].

On the other hand, thanks to the identification of these EGFR mutations, it has been possible to characterize the prevalence of these mutations, which is higher in women, non-smokers, and the Asian population [13].

Administration

Amivantamab is composed so that each vial of 7 ml contains 350 mg of amivantamab at a concentration of 50 mg/ml, with excipients including water for injection, sucrose, polysorbate 80, L-methionine, ethylenediaminetetraacetic acid (EDTA), L-histidine hydrochloride monohydrate. Therefore, this medication is administered via intravenous infusion and is available in vials of 350 mg each. It is crucial to mention that amivantamab has a high potential to induce vomiting and nausea; therefore, it is essential to administer antiemetic medications proactively to prevent these adverse effects [14].

For intravenous administration of amivantamab, it is crucial to adhere to the prescribed infusion rate. The administration process utilizes an infusion set equipped with a flow regulator and a sterile 0.2-micron polyethersulfone filter. This filter is prepared with a low protein-binding diluent. Additionally, due to the potential for infusion-related reactions during the initial treatment phase, it is recommended to administer the drug through a peripheral intravenous line for the first two weeks. After the second week, the drug can be administered through an infusion with a central venous line. It is important to mention that amivantamab should be administered exclusively via a single intravenous route and not concomitantly with other drugs [9].

Dosage

The medication presentation features a concentration of 50 mg/ml (350mg/7ml) for an Injectable Solution. The recommended doses of Rybrevant (Table 2) are administered weekly over a period of four weeks, with the initial dose divided into two infusions during week one, on day one, and day two, followed by administrations every two weeks. Throughout the course of treatment with amivantamab, it is recommended that patients be treated until disease progression is observed while receiving treatment, as it signifies that the treatment is not working and should be discontinued or modified or until unacceptable toxicity (serious side effects) occurs [15].

**Table 2:** Dosage schedule of amivantamab

Weeks	Calendar
From week 1 to 4	Weekly (4 total doses)
After week 5	Every 2 weeks

Given changes in a patient's body weight once treatment has commenced, dose adjustment will not be required. The dosing schedule for amivantamab is described in Table 3 [15].

**Table 3:** Recommended dosage of amivantamab

Body weight of the patient (at reference time) (kilograms)	Recommended dosage (milligrams)	Number of vials
Less than 80 kg	1050 mg	3
Equal to or greater than 80 kg	1400 mg	4

If a dose has not been administered, it should be given as soon as possible, and the dosage schedule should be adjusted while maintaining the recommended treatment interval [15].

Dosage modification

If the patient experiences adverse reactions (AR) of grade 3 or higher, the administration of said medication should be interrupted until the adverse reaction decreases to a lower grade or grade 1. If the medication is suspended for more than seven days, it is important to restart with a low dose (see Table 4 for dose reduction in the case of AR) [16].

**Table 4:** Dose reductions in case of presented adverse reactions

Body weight (at reference time in kilograms)	Initial dose	Dose after the first interruption due to adverse reaction	Dose after the second interruption due to adverse reaction	Third interruption due to an adverse reaction
Less than 80 kg	1050 mg	700 mg	350 mg	Discontinue the medication
More than 80 kg	1400 mg	1050 mg	700 mg	

Recommended concomitant medications

During the first week of treatment, on days one and two prior to starting the infusion, it is recommended to administer drugs such as glucocorticoids, antihistamines, and antipyretics to prevent the risk of infusion-related reactions (IRRs). Following this, for future doses, only the administration of antipyretics and antihistamines is required. Antiemetics are administered as needed [16].

In Table 5, we can observe pre-infusional medication and their respective doses [16].

**Table 5:** Pre-infusional medication, dosage regimen

Pre-infusional	Dosage	Route of administration	Recommended interval of administration before amivantamab administration
Antipyretic (required for all doses)	Paracetamol 650 mg to 1000 mg	Intravenous	15 to 30 minutes
		Oral	30 to 60 minutes
Glucocorticoid (required in the initial dose on days 1 and 2 of week 1, optional in subsequent doses)	Dexamethasone 10 mg or methylprednisolone 40 mg	Intravenous	45 to 60 minutes
Antihistamine (required for all doses)	Diphenhydramine 25 mg to 50 mg	Intravenous	15 to 30 minutes
		Oral	30 to 60 minutes

Preclinical studies

In these preclinical studies, the biomedical community has conducted comprehensive characterization of mutant ErbB proteins, primarily studying wild-type EGFR and ERBB2 proteins, as well as kinase domain mutations. Therefore, many of these preclinical models of isolated proteins or rudimentary cell lines have been instrumental in understanding resistance and response to tyrosine kinase inhibitors (TKIs), along with clinically observed activity of these compounds, as well as new clinical studies [17]. Studies have been conducted that analyze mutations in this receptor and exon 20 insertions.

These exon 20 insertions have been shown to represent a value close to 4 to 12% of EGFR mutations in people with NSCLC [18]. The following mutations, V769_D770insASV, D770_N771insSVD, and A763_Y764insFQEA, correspond to 20%, 19%, and 7% of these mutations, respectively. [19]. This is why the sensitivity of exon 20 insertions to first-generation EGFR-TKIs is low. [20]. The efficacy of these mutations is limited, so the creation of drugs focused on exon 20 insertion is necessary [21]. Therefore, the objective of these findings is to help the development of drugs, as well as the economic analysis and the design and interpretation of clinical trials for new drugs [22].

Clinical studies

Different clinical trials of amivantamab were conducted in NSCLC. The results of significant benefit are associated with the observed objective response rate (ORR) and the duration of responses obtained. These trials demonstrated the reduction in the size of such cancer, and significant beneficial responses were obtained for these patients. Table 6 summarizes the clinical trials of amivantamab reported in NSCLC [23].

**Table 6:** Clinical trials of amivantamab in NSCLC NSCLC - non-small cell lung cancer; SC

Trial	Phase	Setting	Indication	EGFR mutations	Treatment
CHRYSALIS, NCT02609776 [24]	I/IB	Locally advanced or metastatic	Pre-treated NSCLC	Ex20ins	Part 1: Dose escalation of amivantamab as monotherapy or in combination with permetrexed + carboplatin or lazertinib
					Part 2: Cohorts: amivantamab as monotherapy or in combination with pemetrexed + carboplatin or lazertinib
CHRYSALIS-2, NCT04077463 [25]	I/IB	Locally advanced or metastatic	Pre-treated NSCLC	Ex19del, L858R	Phase 1: Dose escalation: lazertinib as monotherapy
					Phase 1b: Amivantamab combination and lazertinib
PAPILLON, NCT04538664 [26]	III	Locally advanced or metastatic	First-line NSCLC	Ex19del, L858R	Pemetrexed + carboplatin vs amivantamab + pemetrexed + carboplatin
MARIPOSA, NCT04487080 [27]	III	Locally advanced or metastatic	First-line NSCLC	Ex19del, L858R, Ex20in (o mutación MET)	Osimertinib vs lazertinib + amivantamab + lazertinib
MARIPOSA2, NCT04988295 [28]	III	Locally advanced or metastatic	Pre-treated NSCLC with osimertinib	Ex19del, L858R, Ex20in	Carboplatin + pemetrexet vs carboplatin + pemetrexed + amivantamab vs carboplatin + permetrexed + lazertinib + amivantamab
PALOMA, NCT04606381 [29]	I	Locally advanced or metastatic	Pre-treated solid tumors	Unknown	Part 1: Amivantamab mixed with SC infusion of rHuPH20 or SC infusion of amivantamab.
					Part 2: SC infusion of high-dose amivantamab or amivantamab formulated with rHuPH20.
					Part 3: Lazertinib + SC infusion of high-dose amivantamab or amivantamab formulated with rHuPH20.
NCT04599712 [30]	EAP (expanded access program)	Locally advanced or metastatic	Pre-treated NSCLC	Ex20ins	Amivantamab + lazertinib
NCT04965090 [31]	II	With metastasis	Pre-treated NSCLC	Ex19del, L858R, Ex20ins	Amivantamab + lazertinib

CHRYSALIS (NCT02609776)

The CHRYSALIS phase I/IB study was designed to assess the safety and efficacy of amivantamab in patients with non-small cell lung cancer, encompassing a total of 362 patients, with a mean age of 62 years and comprising 41% men and 59% women. This study consisted of two parts, the first involving dose escalation of the drug and the second involving dose expansion. It was concluded in the study that the recommended dose of amivantamab is 1050 mg and 1400 mg in patients, and to avoid adverse effects, the first dose should be administered in the first two days of treatment. Furthermore, a reduction in cancer size of approximately 40% was demonstrated in patients, with a duration of 11.1 months and a progression-free survival of 8.3 months, indicating that the treatment proved effective in reducing disease progression and lowering the risk of death [8,24].

CHRYSALIS-2 (NCT04077463)

CHRYSALIS-2 is an open-label study, which includes a single-arm cohort (cohort A) examining patients with EGFR Exon19del or NSCLC L858R whose disease progressed after platinum chemotherapy and in previously treated patients. These patients received the established recommended combined dose of 1050 mg IV of amivantamab (1400 mg, ≥80 kg) + oral lazertinib at a dose of 240 mg. As of April 19, 2021, 116 patients were enrolled in cohort A with an average age of 63 years, 68% female, and 60% Asian, with a median follow-up of 3.7 months, thus 17 patients from the target population, of which 10 remain in treatment with PR and seven with stable disease. The safety profile of cohort A was consistent with previously reported experience with a combined recommended dose of amivantamab plus lazertinib, and no new drug safety signals were identified [25,32]. 

PAPILLON (NCT04538664)

The PAPILLON phase III clinical trial investigates the potential benefits of amivantamab in treating patients with previously untreated, advanced, or metastatic NSCLC harboring Exon20ins mutations. Participants are randomly assigned to receive either the standard-of-care chemotherapy regimen (carboplatin and pemetrexed) alone or the same chemotherapy combined with amivantamab. The current standard treatment for non-squamous NSCLC with Exon20ins mutations is a platinum-based doublet therapy, with the most common combination being carboplatin AUC 5 and pemetrexed 500 mg/m2 administered every 21 days. This trial aims to evaluate whether adding amivantamab to this established therapy can further improve patient outcomes. Patients in the experimental group receive four cycles of the standard chemotherapy followed by maintenance therapy. This maintenance phase involves either pemetrexed alone or pemetrexed combined with amivantamab. Amivantamab is administered as an intravenous infusion, starting with a weekly dose of 1400 mg until the beginning of cycle two. Subsequently, the dose increases to 1750 mg once every 21 days. The primary objective of this large-scale study, involving approximately 300 patients across 200 sites in 25 countries, is to assess progression-free survival. Additionally, the trial design allows patients in the chemotherapy arm to potentially switch to amivantamab monotherapy after a certain point [23,26].

MARIPOSA (NCT04487080)

MARIPOSA is a phase III clinical trial, which enrolls previously untreated patients with advanced or metastatic NSCLC with Ex19del or L858R mutations of EGFR. These patients are randomly selected to receive either lazertinib alone, amivantamab and lazertinib, or the standard treatment osimertinib until progression of the disease. In the case of osimertinib, it is administered at a dose of 80 mg per day according to the Flaura trial, and lazertinib at a dose of 240 mg per day, as identified in the trial. Lazertinib in combination is administered at a dose of 240 mg per day, and amivantamab at a dose of 1050 mg intravenously for body weight less than 80 kg and 1400 mg for those over 80 kg, in 28-day cycles, once weekly with a split dose on days one and two, then every two weeks in subsequent cycles. The endpoint is progression-free survival based on a blind independent central review according to RECIST v1.1 with the accumulation of 1000 patients in 262 sites in 27 countries in total [23,27].

MARIPOSA 2 (NCT04988295)

MARIPOSA 2 is a phase III clinical trial, which enrolls patients with advanced or metastatic NSCLC with Ex19del or L858R EGFR mutations after progression with osimertinib. These patients are randomly assigned to one of three groups: pemetrexed and carboplatin, amivantamab plus pemetrexed and carboplatin, and the combination of amivantamab with lazertinib plus pemetrexed and carboplatin. The endpoint of the trial is progression-free survival according to a blind independent central review using RECIST v1.1 [23,28].

PALOMA

The PALOMA study enrolls patients with different advanced solid tumors, aiming to evaluate the safety, pharmacokinetics, and feasibility of administering amivantamab SC concentration. Cohorts 1a/b and 2a/b received 1050 mg (1400 mg, ≥80 kg), cohort 3a received 1600 mg, and cohort 5a received 2560 mg (3360 mg, ≥80 kg). Cohorts 1 to 3 received weekly doses during the first month and subsequently every two weeks, while cohort 5a received a weekly dose for the first three weeks and then every three weeks. Amivantamab SC was well tolerated with significant results in administration time. The drug provided qualitative and quantitative improvement in symptoms [29,33].

NCT04599712

This other study involved pre-approval access to amivantamab in patients with metastatic non-small cell lung cancer with EGFR exon 20 insertion mutations who have failed platinum-based chemotherapy. Amivantamab is administered intravenously at a recommended dose based on weight of 1050 milligrams or 1400 mg once weekly during the first four-week cycle, which is equivalent to one cycle of 28 days, and then administered every two weeks [23,30].

NCT04965090

The NCT04965090 is a phase II trial, where the combination of drugs amivantamab and lazertinib is being evaluated in patients with central nervous system disease, divided into two experimental arms of patients with brain metastasis and with the leptomeningeal disease with or without metastasis [8,31].

The future treatment of this disease depends on the continuous progress of clinical trials, which is why many exon 20-specific TKIs are under development [34]

Adverse effects

Regarding amivantamab, the most reported adverse effects were skin rash, nail toxicity, constipation, nausea, edema, hypoalbuminemia, and, in some cases, stomatitis and unwanted reactions related to the infusion of the medication. For this reason, antihistamines, corticosteroids, and antipyretics should be administered before the first treatment with the drug to reduce infusion-related reactions [16]. Amivantamab can cause ocular toxicity, including keratitis, dry eye symptoms, conjunctival erythema, visual impairment, mild blurred vision, eye pruritus, and also uveitis [35]. Below is an exemplified summary of the main adverse reactions in patients treated with amivantamab in Table 7 [36].

**Table 7:** Adverse reactions in patients treated with amivantamab

Risk level	Adverse reaction
High risk ≥15%	General disorders and administrative site conditions (17%); Injuries, poisonings, and complications (17%).
Medium risk 5-15	Gastrointestinal disorders (5%); Infections and infestations (7%); Benign neoplasms (6%)
Low risk ≤5%	Blood and lymphatic system disorders (1%); Cardiac disorders (2%); Eye disorders (1%); Immune system disorders (1%); Metabolism and nutrition disorders (1%)

Precautions

During pregnancy, exposure to amivantamab, based on animal model data, has been reported to have the potential to cause fetal harm. Therefore, its use during pregnancy is not recommended. Regarding breastfeeding, data cannot confirm if the drug is secreted in breast milk; however, it is not recommended to breastfeed during the treatment period but rather until three months after the last dose of the drug. In the geriatric population, no differences in safety or efficacy are observed between older adults and regular patients of younger age [8].

## Conclusions

It can be concluded that amivantamab represents a major breakthrough for patients with EGFR exon 20 insertion mutations in lung cancer. This bispecific antibody fills a critical gap by offering clinically meaningful efficacy where there were previously limited options. It demonstrates improved efficacy and tolerability compared to other treatments for this condition due to its unique dual targeting of EGFR and MET and its well-tolerated structure. While the CHRYSALIS trial showed promising response rates and safety, longer-term monitoring is needed to definitively confirm its effectiveness, but the initial data suggests amivantamab offers a durable and well-tolerated treatment option for this patient population.

## References

[1] Abal Arca J, Parente Lamelas I, Almazán Ortega R, Blanco Pérez J, Toubes Navarro ME, Marcos Velázquez P (2009). Lung cancer and COPD: a common combination (Article in Spanish). Arch Bronconeumol.

[2] Rudin CM, Brambilla E, Faivre-Finn C, Sage J (2021). Small-cell lung cancer. Nat Rev Dis Primers.

[3] Amorín Kajatt E (2013). Lung cancer: a review of current knowledge, diagnostic methods and therapeutic perspectives (Article in Spanish). Rev Peru Med Exp Salud Publica.

[4] Maemondo M, Inoue A, Kobayashi K (2010). Gefitinib or chemotherapy for non-small-cell lung cancer with mutated EGFR. N Engl J Med.

[5] Sequist LV, Yang JC, Yamamoto N (2013). Phase III study of afatinib or cisplatin plus pemetrexed in patients with metastatic lung adenocarcinoma with EGFR mutations. J Clin Oncol.

[6] Neijssen J, Cardoso RM, Chevalier KM (2021). Discovery of amivantamab (JNJ-61186372), a bispecific antibody targeting EGFR and MET. J Biol Chem.

[7] Brazel D, Nagasaka M (2021). Spotlight on amivantamab (JNJ-61186372) for EGFR exon 20 insertions positive non-small cell lung cancer. Lung Cancer (Auckl).

[8] Petrini I, Giaccone G (2022). Amivantamab in the treatment of metastatic NSCLC: patient selection and special considerations. Onco Targets Ther.

[9] John A, Noronha V, Singh A, Menon N, Prabhash K (2023). Amivantamab: a narrative drug review. Cancer Res Stat Treat.

[10] Vyse S, Huang PH (2022). Amivantamab for the treatment of EGFR exon 20 insertion mutant non-small cell lung cancer. Expert Rev Anticancer Ther.

[11] Ryszkiewicz P, Malinowska B, Schlicker E (2023). Polypharmacology: promises and new drugs in 2022. Pharmacol Rep.

[12] Minchom A, Viteri S, Bazhenova L (2022). Amivantamab compared with real-world therapies in patients with advanced non-small cell lung cancer harboring EGFR exon 20 insertion mutations who progressed after platinum-based chemotherapy. Lung Cancer.

[13] Mitsudomi T, Yatabe Y (2007). Mutations of the epidermal growth factor receptor gene and related genes as determinants of epidermal growth factor receptor tyrosine kinase inhibitors sensitivity in lung cancer. Cancer Sci.

[14] Krebs M, Johnson ML, Cho BC (2021). Subcutaneous delivery of amivantamab in patients with advanced solid malignancies: PALOMA, an open-label, multicenter, dose escalation phase 1b study. Develop Therap Molecul Target Agents Tumor Biol.

[15] Castaño C (2023). National executive power. ANMAT.

[16] Janssen: Rybrevant® f.f (2022). Infusion solution concentrate monograph. Infusion Solution Concentrate Monograph.

[17] Statements CCF-L (2024). Johnson & Johnson receives positive CHMP opinion for RYBREVANT® (amivantamab) in combination with chemotherapy for the first-line treatment of patients with advanced non-small cell lung cancer with activating EGFR exon 20 insertion mutations. https://www.jnj.com/media-center/press-releases/johnson-johnson-receives-positive-chmp-opinion-for-rybrevant-amivantamab-in-combination-with-chemotherapy-for-the-first-line-treatment-of-patients-with-advanced-non-small-cell-lung-cancer-with-activating-egfr-exon-20-insertion-mutations.

[18] Arcila ME, Nafa K, Chaft JE (2013). EGFR exon 20 insertion mutations in lung adenocarcinomas: prevalence, molecular heterogeneity, and clinicopathologic characteristics. Mol Cancer Ther.

[19] Kobayashi Y, Mitsudomi T (2016). Not all epidermal growth factor receptor mutations in lung cancer are created equal: perspectives for individualized treatment strategy. Cancer Sci.

[20] Yasuda H, Park E, Yun CH (2013). Structural, biochemical, and clinical characterization of epidermal growth factor receptor (EGFR) exon 20 insertion mutations in lung cancer. Sci Transl Med.

[21] Yang GJ, Li J, Xu HY (2021). Osimertinib for Chinese advanced non-small cell lung cancer patients harboring diverse EGFR exon 20 insertion mutations. Lung Cancer.

[22] Lee CK, Wu YL, Ding PN (2015). Impact of specific epidermal growth factor receptor (EGFR) mutations and clinical characteristics on outcomes after treatment with EGFR tyrosine kinase inhibitors versus chemotherapy in EGFR-mutant lung cancer: a meta-analysis. J Clin Oncol.

[23] Sentana-Lledo D, Academia E, Viray H, Rangachari D, Kobayashi SS, VanderLaan PA, Costa DB (2023). EGFR exon 20 insertion mutations and ERBB2 mutations in lung cancer: a narrative review on approved targeted therapies from oral kinase inhibitors to antibody-drug conjugates. Transl Lung Cancer Res.

[24] Janssen Research & Development, LLC LLC (2024). A Phase 1, first-in-human, open-label, dose escalation study of JNJ-61186372, a Human Bispecific EGFR and cMet Antibody, in subjects with advanced non-small cell lung cancer. clinicaltrials.gov.

[25] Janssen Research & Development (2024). 1b study to evaluate the safety and pharmacokinetics of JNJ-73841937 (lazertinib), a third generation EGFR-TKI, as monotherapy or in combinations with JNJ-61186372, a Human Bispecific EGFR and cMet Antibody in participants with advanced non-small cel. clinicaltrials.gov.

[26] Janssen Research & Development, LLC LLC (2024). A randomized, open-label Phase 3 study of combination amivantamab and carboplatin-pemetrexed therapy, compared with carboplatin-pemetrexed, in patients with EGFR exon 20ins mutated locally advanced or metastatic non-small cell lung cancer. clinicaltrials.gov.

[27] Janssen Research & Development, LLC LLC (2024). A phase 3, randomized study of amivantamab and lazertinib combination therapy versus osimertinib versus lazertinib as first-line treatment in patients with EGFR-mutated locally advanced or metastatic non-small cell lung cancer. clinicaltrials.gov.

[28] Janssen Research & Development, LLC LLC (2024). A phase 3, open-label, randomized study of amivantamab and lazertinib in combination with platinum-based chemotherapy compared with platinum-based chemotherapy in patients with EGFR-mutated locally advanced or metastatic non-small cell lung cancer AF. clinicaltrials.gov.

[29] Janssen Research & Development (2024). LLC: An open-label, multicenter, dose escalation Phase 1b study to assess the safety and pharmacokinetics of subcutaneous delivery of Amivantamab, a Human Bispecific EGFR and cMet antibody for the treatment of advanced solid malignancies. clinicaltrials.gov.

[30] Janssen Research & Development (2021). LLC: Pre-approval access with amivantamab in patients with metastatic non-small cell lung cancer with EGFR exon 20 insertion mutations who have failed platinum-based chemotherapy. clinicaltrials.gov.

[31] Memorial Sloan Kettering Cancer Center (2023). A Phase 2 single-arm study of amivantamab (JNJ-61186372) and lazertinib in metastatic EGFR-mutant lung cancer with progressive or new CNS metastases on previous treatment. clinicaltrials.gov.

[32] Chon K, Larkins E, Chatterjee S (2023). FDA approval summary: Amivantamab for the treatment of patients with non-small cell lung cancer with EGFR exon 20 insertion mutations. Clin Cancer Res.

[33] Shu C, Goto K, Ohe Y (2021). 1193MO Amivantamab plus lazertinib in post-osimertinib, post-platinum chemotherapy EGFR-mutant non-small cell lung cancer (NSCLC): Preliminary results from CHRYSALIS-2. Annals of Oncology.

[34] (2023). Targeted oncology: Precise management of EGFR exon 20-positive non-small cell lung cancer. https://www.targetedonc.com/view/precise-management-of-egfr-exon-20-positive-non-small-cell-lung-canc-er.

[35] Bazhenova L, Minchom A, Viteri S (2021). Comparative clinical outcomes for patients with advanced NSCLC harboring EGFR exon 20 insertion mutations and common EGFR mutations. Lung Cancer.

[36] (2024). World Health Organization: reported potencial side effects amivantamab. https://www.vigiaccess.org/.

